# Bovine Tuberculosis in Doñana Biosphere Reserve: The Role of Wild Ungulates as Disease Reservoirs in the Last Iberian Lynx Strongholds

**DOI:** 10.1371/journal.pone.0002776

**Published:** 2008-07-23

**Authors:** Christian Gortázar, María José Torres, Joaquín Vicente, Pelayo Acevedo, Manuel Reglero, José de la Fuente, Juan José Negro, Javier Aznar-Martín

**Affiliations:** 1 IREC National Wildlife Research Institute (CSIC-UCLM-JCCM), Ciudad Real, Spain; 2 Departamento de Microbiología, Universidad de Sevilla, Sevilla, Spain; 3 Department of Veterinary Pathobiology, Center for Veterinary Health Sciences, Oklahoma State University, Stillwater, Oklahoma, United States of America; 4 Department of Evolutionary Ecology, Estación Biológica Doñana, CSIC, Sevilla, Spain; 5 Servicio de Microbiología, HH UU Virgen del Rocío, Sevilla, Spain; University of Kent, United Kingdom

## Abstract

Doñana National Park (DNP) in southern Spain is a UNESCO Biosphere Reserve where commercial hunting and wildlife artificial feeding do not take place and traditional cattle husbandry still exists. Herein, we hypothesized that *Mycobacterium bovis* infection prevalence in wild ungulates will depend on host ecology and that variation in prevalence will reflect variation in the interaction between hosts and environmental risk factors. Cattle bTB reactor rates increased in DNP despite compulsory testing and culling of infected animals. In this study, 124 European wild boar, 95 red deer, and 97 fallow deer were sampled from April 2006 to April 2007 and analyzed for *M. bovis* infection. Modelling and GIS were used to identify risk factors and intra and inter-species relationships. Infection with *M. bovis* was confirmed in 65 (52.4%) wild boar, 26 (27.4%) red deer and 18 (18.5%) fallow deer. In the absence of cattle, wild boar *M. bovis* prevalence reached 92.3% in the northern third of DNP. Wild boar showed more than twice prevalence than that in deer (p<0.001). Modelling revealed that *M. bovis* prevalence decreased from North to South in wild boar (p<0.001) and red deer (p<0.01), whereas no spatial pattern was evidenced for fallow deer. Infection risk in wild boar was dependent on wild boar *M. bovis* prevalence in the buffer area containing interacting individuals (p<0.01). The prevalence recorded in this study is among the highest reported in wildlife. Remarkably, this high prevalence occurs in the absence of wildlife artificial feeding, suggesting that a feeding ban alone would have a limited effect on wildlife *M. bovis* prevalence. In DNP, *M. bovis* transmission may occur predominantly at the intra-species level due to ecological, behavioural and epidemiological factors. The results of this study allow inferring conclusions on epidemiological bTB risk factors in Mediterranean habitats that are not managed for hunting purposes. Our results support the need to consider wildlife species for the control of bTB in cattle and strongly suggest that bTB may affect animal welfare and conservation.

## Introduction

Bovine tuberculosis (bTB) due to *Mycobacterium bovis* and closely related bacterial strains of the *M. tuberculosis* complex is a worldwide disease that affects a wide range of domestic and wildlife animals and humans. Nowadays bTB has become a major sanitary and conservation problem for different reasons even in relatively unmanaged natural areas across the world (e.g. Kruger National Park, South Africa; Doñana National Park (DNP), Spain; Riding Mountain National Park and Wood Buffalo National Park, Canada; Queen Elizabeth National Park, Uganda). Similar to other diseases shared with wildlife [1] the existence of wildlife reservoirs is limiting the effectiveness of bTB eradication schemes largely based on testing and culling of infected cattle [2]. As a consequence, there is a need to better integrate conservation biology with livestock policy to develop management options [3].

In southern Spain, an increase in *M. bovis* prevalence in wild ungulates has been reported [4], and large-scale data show mean macroscopic bTB-compatible lesion prevalence of 14% in red deer (*Cervus elaphus*) and 42% in European wild boar (*Sus scrofa*). Wild boar bTB prevalence has been found to correlate with prevalence in red deer, although wild boar always showing higher prevalence than deer [5]. Recent pathological and epidemiological evidence strongly suggests that the wild boar is a true *M. bovis* reservoir in Mediterranean Spain [6], [7]. Known risk factors for bTB in wild boar include increasing age [5], [8], density and spatial aggregation [9], particularly at waterholes [8], fencing and genetic factors [10], and other habitat features [8]. Risk factors for bTB in red deer also include increasing age [5], gender, males being more susceptible than females, and wild boar spatial aggregation at both feeding and watering places [8], [11]. Indications of genetic factors affecting bTB susceptibility in Iberian red deer have recently been found [12]. In addition, artificial feeding of deer has been suggested as a bTB risk factor in Spain [11] and elsewhere [13].

Fallow deer (*Dama dama*) are more gregarious than other European deer species and bTB has been reported in fallow deer from Spain [2], but little is known regarding disease risk factors in free living fallow deer. In fact, most reports of bTB in this deer species refer to captive or farmed deer rather than free-ranging populations (e.g. [14]). Thus, this is to the best of our knowledge the first epidemiological study on *M. bovis* infection in wild fallow deer.

DNP in southern Spain is a UNESCO Biosphere Reserve and one of the two last strongholds for the Iberian lynx (*Lynx pardinus*), a highly endangered felid that is susceptible to bTB [15]. DNP also represents an important conservation “island” for a rich community of vertebrates. In contrast to most other Spanish wild ungulate populations, commercial hunting and artificial wildlife feeding do not take place in DNP, and traditional cattle husbandry still exists. This, along with the direct threat for the survival of endangered lynx makes DNP an ideal and complex study area for bTB ecology. Little information is available, but *M. bovis* probably entered the Park historically through domestic cattle, which have been herded in the area for centuries [16]. To our knowledge, the first bTB cases described in wild ungulates from Doñana were reported in the 1980s [17]. According to internal reports of the Veterinary Services of Doñana National Park, bTB cases have consistently been reported from Doñana wildlife since 1993. Prevalences of macroscopic lesions compatible with tuberculosis were 87% in 25 wild boar and 26% in 33 deer (red and fallow deer pooled) in 1993. An increase in *M. bovis* infection prevalence was observed between 1996 and 1998 [18]. From 1998 to 2003, a sample of 214 wild boar, 168 red deer and 134 fallow deer (age, sex and sampling site unknown) yielded *M. bovis* infection prevalences of 28%, 15% and 13%, respectively. Molecular characterization revealed that wildlife and cattle share the same strains [19]. However, the long-term impact of bTB on free-ranging wildlife populations for DNP is unknown.

Herein, we hypothesized that *M. bovis* infection prevalence in wild ungulates will depend on host ecology and that variation in prevalence will reflect variation in the interaction between hosts and environmental risk factors. For example, if close contact between individuals or contact with livestock is a key factor in *M. bovis* transmission, one would expect the gregarious fallow deer to display the highest *M. bovis* infection prevalence, and those areas shared between wildlife and cattle to have higher prevalences than areas with wildlife alone. We also expected *M. bovis* infection prevalence to be higher in areas of higher wildlife abundance or higher spatial aggregation, such as those where shallow sweet water points remain scarcer during the dry season in summer. By identifying the possible reasons for those eventual spatial and inter-specific differences, we aimed to infer management implications of interest not only for DNP but also for a broader range of wildlife bTB situations in, at least, Mediterranean areas.

## Materials and Methods

### Study area

**Figure 1:**
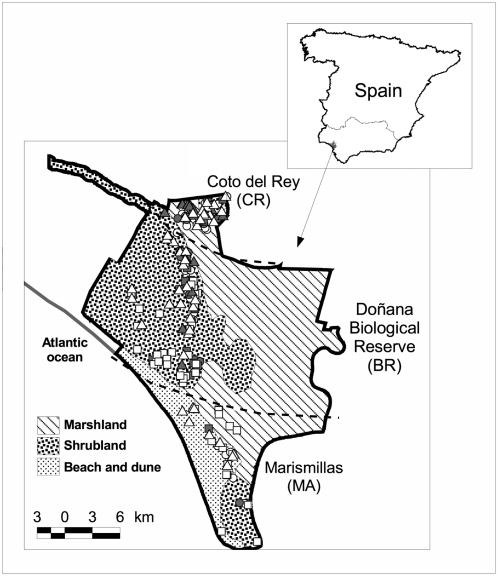
Map of Doñana National Park and the sampling sectors. Sampled wild boar (squares) fallow deer (circles) and red deer (triangles) are shown. Dark symbols mean *Mycobacterium bovis* culture positive samples and open symbols mean negative ones.

The study was performed in the DNP (Figure 1) in south-western Spain (37°0′N, 6°30′W), covering an area of 54.252 ha in the Andalusian provinces of Cádiz and Huelva. This is a flat region of sandy soils bordering on the Atlantic Ocean, with altitudes ranging 0–47 m a.s.l. The climate is sub-humid Mediterranean and seasonality is strong, especially regarding the water level and its effects on the vegetation. In the wet season (winter and spring), most of the marshlands are flooded and wildlife and cattle concentrate in the slightly more elevated scrublands. In summer, the ecotone between the scrublands and the marshes produces aggregation of wild and domestic ungulates. DNP is one of the most important natural reserves in Europe in terms of biodiversity. Human access is restricted and management is carried out by Conservation authorities. Limited traditional exploitation of some natural resources, such as pine-cone collection and cattle and horse rising are allowed [20]. As part of the Park management, culling of ungulate populations by park rangers is practised. This culling is mainly addressed to the wild boar, of which several hundreds may be eliminated each year (10–20% of the estimated population).

Three different geographic areas along a north-south gradient can be identified in DNP (see Figure 1). To the north, Coto del Rey (CR) is characterized by pine forests (*Pinus pinea*) and Mediterranean scrubland patches of *Pistacia lentiscus* and *Halimium halimifolium* with scattered *Quercus suber* trees and *Fraxinus angustifolius* flanking seasonal streams. At the centre, Doñana Biological Reserve and its surroundings (BR) include the heart of DNP. The vegetation is mainly heterogeneous Mediterranean scrubland, a product of more traditional land usage by humans, dominated by *H. halimifolium*, *Ulex* spp. *Erica* spp. and *Juniperus phoenicea*. To the south, Marismillas (MA) has an important proportion of sand dune habitat, marshland and pine forests, along with patches of Mediterranean scrubland.

### Cattle

Doñana cattle are a mix of breeds, including beef cattle such as Charolais and Limousin, and autochthonous breeds such as Retinta and Mostrenca. Cattle are nowadays evenly distributed in the study area but for CR, where no cattle are allowed due to lynx conservation needs, as a means to maximize pasture availability for its staple prey wild rabbits (*Oryctolagus cuniculus*). Nonetheless, CR used to be the area with highest cattle densities prior to 2005. CR cattle density in 2002 was 24 per sq. km and total park cattle density was 7 per sq. km in that year. The number of cattle has been reduced by about 50% from the first survey in 1979 to present. These animals, numbering about 1600 nowadays, belong to 300 different owners and are kept together in 9 adjacent enclosures of a similar area that cover the entire DNP but CR. Cattle move freely within but not among enclosures, whereas wildlife has no problem to cross the fences.

**Figure 2:**
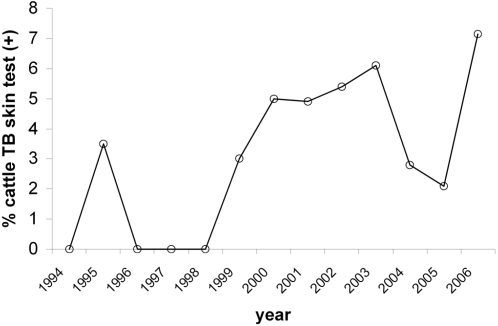
Annual percentage of skin test bovine tuberculosis (bTB) reactor rates among Doñana cattle from 1994 to 2006.

In 2006, skin testing of the 1577 cattle from the DNP study area revealed a rate of 9.4% bTB reactors (data from Veterinary Services). Despite compulsory annual testing and subsequent culling, bTB reactor rates have been steadily increasing between 1994 and 2006 (Figure 2), and wildlife is blamed as a reservoir. Reactors were detected throughout the study area. Slaughterhouse sampling of the retropharyngeal lymph nodes of 98 of these cattle in 2006 allowed isolation of *M. bovis* in 47 cases. Molecular typing (MIRU_VNTR and Spoligotyping) confirmed that the same strains circulate among cattle and wildlife (data not shown).

### Wildlife

Population estimations of wild ungulates are available for deer from 1986 (http://www-rbd.ebd.csic.es/Seguimiento/mediobiologico.htm) and for wild boar in 1994 [16] and in 2006 (http://www-rbd.ebd.csic.es/Seguimiento/mediobiologico.htm). Total autumn population size in 2006 was estimated at 603 red deer, 790 fallow deer, and 1700 European wild boar. Deer and wild boar abundances are higher in CR than in MA, with BR representing an intermediate situation where local aggregations occur at the more productive grassy ecotones between the marshes and scrublands (see, e.g., [21]). This remains as a moist area all year round with green pasture available for grazing animals. About 50 lynx remain nowadays in DNP, feeding mostly on rabbits but occasionally on wild ungulates [22], [23].

### Animal sampling

From April 2006 to April 2007, 124 European wild boar, 95 red deer, and 97 fallow deer were sampled within the park by shooting. A necropsy was performed in the field, including detailed determination of morphometry, weight, and sex. Based on tooth eruption patterns, animals less than 12 months old were classified as juveniles, those between 12 and 24 months as yearlings, and those more than 2 years old as adults [24].

### Presence of tuberculosis-like lesions

In deer, bTBL were diagnosed by necropsy of the entire animal with detailed macroscopic inspection of lymph nodes and abdominal and thoracic organs. This examination routinely included parotid, retropharyngeal and submandibular lymph nodes in the head, tracheobronchial and mediastinal lymph nodes and lungs in the thorax, and hepatic and mesenteric lymph nodes, ileocecal valve, kidneys, liver and spleen in the abdomen. Gross lesions in other locations were also recorded. In wild boar, in which no thoracic or abdominal viscera were inspected due to permit constraints, lymphoid tissues of the head alone were considered useful for diagnosis [6], [25]. Lymph nodes were dissected, sectioned serially and carefully examined for gross lesions.

### Pathology

Tissue samples from the aforementioned collected LNs, the diaphragmatic lung lobes, the kidneys, adrenals, liver, spleen, ileocaecal valve and any other bTB compatible lesion were fixed in 10% neutral buffered formalin and routinely processed in order to obtain haematoxylin and eosin (HE) and Ziehl–Neelsen (Z–N) stained slides. Gram and PAS stains were performed to discard non mycobacterial granulomas [6].

### Laboratory procedures

In wild boar, two pieces of the tonsils and a pool of samples from both mandibular lymph nodes were submitted for microbiology. In deer, two pieces of the tonsils and a pool of head lymph node samples, always containing at least half left and half right medial retropharyngeal lymph node, were submitted to culture. All microbiology was done at the Virgen del Rocío Hospital in Seville, in a laboratory meeting biosecurity level 3 requirements. Briefly, 2 g samples were decontaminated adding an equivalent volume of 2% N-acetil-L-cistein-NaOH, vortexed, and incubated at room temperature for 15 minutes. The reaction was stopped with phosphate buffer (pH 6.8), to a total volume of 50 mL. Samples were than centrifuged at 3.000× g for 15 minutes. The sediment was re-suspended in 5 mL of sterile water. Each of four tubes of Löwenstein-Jensen piruvate medium was inoculated with three drops of the homogenate incubated at 37°C and inspected weekly with the aid of a stereoscopic microscope for the presence of any growth, until 8 weeks old. The colonies were identified by standard bacteriological methods and identification was confirmed by a DNA probe system (Genotype®MTBC, Hain Lifescience GmbH, Germany). Samples which yielded mycobacteria other than *M. bovis* further were excluded of this study (data not shown).

### Statistics

The chi square test was used to compare prevalence between species. Confidence intervals for prevalences were estimated by the expression S.E.95%C.I. = 1.96[p(1-p)]/n1/2 [26].

We tested the factors affecting the risk of *M. bovis* infection by means of generalized linear models. The response variable was the *M. bovis* culture status (as categorical binomial, 0 = negative, 1 = positive) for each species (wild boar, red deer and fallow deer). Sex as categorical, and age and UTM coordinates (X and Y) as continuous were included as explanatory variables.

In order to reveal intra and inter-specific relationships in the pattern of *M. bovis* infection, we secondly tested the statistical associations between the individual risk of culturing positive to *M. bovis* (separately for each species) and the prevalence exhibited by populations of wild boar, red deer and fallow deer surrounding the given individual. For this purpose, we quantified and mapped the prevalence of *M. bovis* culture in a 522.8 ha buffer area (1290 m of radius) surrounding each individual using ArcGis vs. 9.1. We selected this buffer area since it includes the maximum home range area described for either of our host species in Mediterranean habitats (337.8±184.8 ha for red deer in DNP [27]; 337.5±178.9 ha for fallow deer in Italy [28]; 400 ha for female wild boar in Italy [29]. The results were included as an explanatory variable in 3 different models for each species (comparing against individuals of the same species, and the other two, respectively) where the individual risk of culturing positive *M. bovis* was always the binary response variable. Models also included sex, age and coordinates as previously. Up to two degree interactions were included. Individuals with no sampled animals within their buffer areas were not included in the analyses (sample sizes were n = 118, 66 and 64 for the models including wild boar as response variable and buffers of wild boar, red deer and fallow deer, respectively; n = 73, 90, and 78 for red deer as response variable, and n = 78, 86, 98 for fallow deer as response variable). We always modelled with a binomial link function and a logit error. Statistical analyses were performed with SPSS 15.0 (SPSS Inc. 2006). Intervals are shown with standard error.

## Results

Out of the total sample tested (n = 316), 86 wild ungulates were sampled in the smaller northern third of the park (CR), 172 in BR, and 58 in the southern third (MA). The low sample size in MA was due to limitations imposed by the DNP management, based on lower deer abundances in this southern portion of the park.

**Figure 3:**
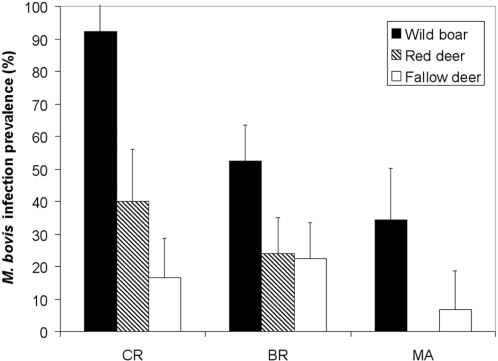
*Mycobacterium bovis* infection prevalence (in %+95% CI) in European wild boar, red deer and fallow deer from Doñana National Park, Spain. The north (CR) to south (MA) gradient in bTB prevalence is most evident in the wild boar.

*M. bovis* was isolated from tissue samples of 65 wild boar, 52.4% (95%CI 43.6–61.2), 26 red deer, 27.4% (18.4–36.3), and 18 fallow deer, 18.5% (10.8–26.3). The inter-species differences in *M. bovis* infection prevalence were significant (*Chi* ^2^ = 30.7, 2 d.f., p<0.001), wild boar showing more than twice than that in deer. The models testing the effect of location revealed that a North-South gradient (Y coordinates) was significant for wild boar (*Chi* ^2^ = 11.57, 1 d.f., p<0.001) and red deer (*Chi* ^2^ = 6.65, 1 d.f., p<0.01), decreasing the prevalence from the North to the South (Figure 1), whereas no spatial pattern was evidenced for fallow deer. Surprisingly wildlife from CR (the only currently cattle-free area) had the highest prevalence (Figure 3).In this northern third of DNP, *M. bovis* infection prevalence was still high among yearlings (3 out of 3 wild boar, 100%; 3/5 red deer, 60%; 1/9 fallow deer, 11%).

The models testing the statistical relationships between species revealed that the infection risk for wild boar was dependent on the wild boar *M. bovis* infection prevalence in the buffer area (*Chi* ^2^ = 10.15, 1 d.f., p<0.01). Also, age (*Chi* ^2^ = 4.24, 1 d.f., p = 0.04) and its interaction with wild boar infection prevalence in the buffers (*Chi* ^2^ = 8.95, 1 d.f., p<0.01) were statistically significant, displaying an age decreasing pattern towards adult age (adult wild boar 43.1%, juveniles 65.5% and yearlings 65.2%), and being more marked the effect of wild boar prevalence on individual *M. bovis* infection status in younger ages than in adults (wild boar buffer prevalences were 29.16±22.73 and 70.66±11.76 for negative and positive juveniles, respectively; 33.85±14.96 and 67.16±10.77 for negative and positive yearlings, respectively, and 42.97±7.73 and 53.51±8.96 for negative and positive adults, respectively). No inter or intra-specific relationships were revealed in the models for red deer and fallow deer, respectively.

Exactly 50% of all *M. bovis* culture-positive deer showed generalized bTB lesions, affecting more than one anatomic region and most frequently all three (head, thorax and abdomen). In wild boar only tonsils and mandibular lymph nodes were inspected. Hence, no estimation of the proportion of wild boar with generalized bTB was possible.

## Discussion

Data obtained in this study provide insight into bTB epidemiology in a free living, mixed ungulate population, and allow inferring conclusions on epidemiological bTB risk factors in Mediterranean habitats that are not managed for hunting purposes. Our results support the need to consider wildlife in any attempt to control diseases of domestic livestock such as bTB [1]. Furthermore, data reported in this study strongly suggest that bTB may affect animal welfare as well as conservation efforts in the DNP area. Since this was a correlational study, we will consider our conclusions as exploratory but very relevant to infer management implications of interest in Mediterranean areas.

### Prevalence of *M. bovis* infection in wildlife species

The *M. bovis* infection prevalence recorded in this study, especially in wild boar, is among the highest of the literature in wildlife populations. As a comparison, prevalences higher than 30% are rarely found in the badger [30], [31] and in New Zealand possums [32]. In deer species, the highest prevalence ever recorded was described in New Zealand, where an acute outbreak at prevalence higher than 90% in young fawns occurred in a farm [33]. In wild deer from New Zealand a prevalence of 37% was reported [34]. Prevalences estimated in wild deer populations in the northern hemisphere were always below 6% [35]. Only one study on wild ungulates from south – central Spain (mainly focused on fenced hunting estates) detected higher local prevalence than those reported in DNP (up to 50% of red deer, mean 14% [5]. In the case of wild boar, the high disease prevalence based on culture observed in DPN is remarkable [7]. Enzootic *M. bovis* infection has previously been reported in wild boar in Italy (prevalence ranging up to 15% [36], [37], France (37.5% based on isolation [38]) and in other countries from central and western Europe (in Germany, 1.4% based on gross lesions, and in 7 other countries [39]). In New Zealand, local prevalences as high as the ones in our study have been found in feral pigs (reviewed in [40]). Recently, macroscopic bTB-compatible lesions were detected in up to 100% of the sampled wild boar (mean 42%) in Central Spain [5].

In DNP, all three wild ungulate species showed higher *M. bovis* infection prevalences than those detected by compulsory skin test in local free ranging cattle. The fact that the percentage of skin test reactor rates among cattle increased in DNP despite annual test and slaughter shows that, at least in the DNP, current cattle bTB control is not enough to eradicate the disease. Moreover, the highest *M. bovis* infection prevalence in wild ungulates was found in the northern area of DNP (CR), a section of the park where cattle have been excluded since 2005. The high *M. bovis* infection prevalences among yearlings from CR suggest a still high infection pressure. Thus, wildlife has probably been able to maintain *M. bovis* circulation in the absence of livestock for at least 3 years in this area, confirming previous observations from south-central Spain [41]. Though sampling and methodological differences need to be considered, our results also reveal an increase in *M. bovis* infection prevalence as compared to data from 1998 to 2003 [19].

### Bovine tuberculosis risk factors

In a large scale survey of mainly private hunting estates in south-central Spain, infection levels in both red deer and wild boar, spatial aggregation at waterholes, and artificial game management including fencing and feeding, were identified as bTB risk factors for wild ungulates [5], [8], [10], [11]. Few of the sampling sites included in the south-central Spain study were still grazed by cattle, and hence only limited information on the risk posed by wildlife to cattle, or vice versa, was obtained. In contrast, cattle and wild ungulates share the pastures and waterholes of DNP, and both direct and indirect contacts must be frequent. Molecular typing of *M. bovis* strains from wildlife and cattle in DNP confirmed the circulation of the same strains in both hosts [19]. However, a detailed study on contact rates and factors favouring disease transmission between cattle and wildlife is needed to clarify how this transmission takes place, and to eventually identify means to limit this risk.

DNP is also interesting because, in contrast to many hunting estates in Spain, no wildlife management such as artificial feeding takes place. It is clear that spatial aggregation, even more than density, is a major risk factor for bTB in wildlife (e.g. [9]). In fact, banning wildlife feeding has been proposed as a means to limit direct or indirect *M. bovis* transmission at feeding sites, for instance in white-tailed deer (*Odocoileus virginianus*) from Michigan [42]. However, in warm climates with marked dry seasons, waterholes may be as much a risk factor as feeding sites. This is supported by the survey on wildlife bTB in south-central Spain [11]. The extremely high prevalence found in DNP in the absence of artificial feeding of wildlife, suggests that a feeding ban alone would have limited effect on wildlife *M. bovis* prevalences in Mediterranean habitats. Watering practices performed in the northern area of DNP may relate to the spatial pattern we found (see below).

### Differences in bTB prevalence between species

Wild boar consistently showed higher *M. bovis* infection prevalence than deer in DNP. This was already reported in other studies on bTB in Spain [4], [5]. Epidemiological studies in Spain suggest that wild boar get more easily infected than red deer [5]. Vicente et al. [11] suggested that management factors, which maintain high densities and spatially concentrate wild ungulates, may increase the frequency and probability of both direct and indirect transmission, and this could primarily affect wild boar. Nevertheless, our study populations are characterized by a lack of management. One possible explanation is that the wild boar is a carrion consumer and becomes infected by feeding on tuberculous carrion [43]. To confirm this fact in DNP, in spring 2006 we placed ten shot fallow deer in the field until consumption by scavengers. The carcasses were monitored with infrared-activated digital cameras. All carcasses were consumed by wild boar, alone or in large packs including females and lactating piglets (unpublished results). Thus, infection through consumption of contaminated carcasses may increase the probability of wild boar contacting *M. bovis* as compared to deer species. Nevertheless, an increasing availability of infected carcasses and the subsequent increase in *M. bovis* infection prevalence in wild boar is expected to be found in areas where bTB is highly prevalent in deer, but we failed to identify any interspecific interaction between these factors.

To our knowledge, this is the first detailed bTB survey on wild fallow deer and red deer sharing the same area. Our observational data do not allow inferring the reasons for the lower prevalence in fallow deer as compared to red deer. In fact, fallow deer are even more gregarious than red deer [44], and we expected to observe an inverse prevalence figure. The percentage of infected animals showing generalized bTB did not differ between fallow deer and red deer, which suggests that, rather than a matter of resistance, it could be due to exposure. For example, Munro [45] showed that fallow deer and sika deer (*Cervus nippon*) are more susceptible to *M. avium* infections than red deer. An opposite situation could apply for *M. bovis*, although this needs to be confirmed. Differences in grazing patterns somehow could determine a different exposition to mycobacteria since the red deer is more a browser than the fallow deer. In this sense, hardwood *Quercus* spp. forest availability was associated with increased bTB risk in red deer and wild boar in central Spain [5]. Miller et al. [13] suggested that woodland areas provide shady, moist conditions under which *M. bovis* might survive longer in the environment. Scrublands and woodlands, which are preferably used by red deer compared with fallow deer, could provide the best available environment for mycobacteria persistence in Mediterranean habitats, where rainfall is highly variable amongst seasons, but retention of moisture in woodlands may favour the environmental persistence of mycobacteria. If environmental contamination exists, red deer feeding in the area (by muzzling) could either ingest or inhale mycobacteria. Also, it is well known that red deer rutting in DNP mainly occurs in the ecotone between the marsh and the scrublands [21], which could provide improved conditions for mycobacteria transmission. Finally, a differential use and aggregation of deer species around watering areas, especially during summer time, could also cause these differences between species.

Evidences of inter-species associations in the pattern of *M. bovis* infection were not found. Evidence consistent with the wild boar being a potential *M. bovis* reservoir in Central Spain includes the observations that in several populations where red deer were absent, bTBL were found in wild boar, and the MTB complex was isolated [5], [41]. Therefore, although inter-species transmission probably occurs (e.g. see above concerning scavenging), the predominant transmission may occur at the intra-species level due to ecological, behavioural and epidemiological factors here discussed. Behavioural studies and molecular analyses of *M. bovis* strains could provide insight into these patterns found across deer species.

Finally, species-to-species differences in the response to *M. bovis* infection have been reported in naturally infected wild boar and deer populations [12], [46], [47]. Differential gene and protein expression in *M. bovis*-infected individuals may correlate with resistance to bTB and therefore could also affect disease prevalence in different species [48], [49].

### The north-south gradient of *M. bovis* infection prevalence

Another latitudinal gradient of *M. bovis* infection prevalence occurs in Krüger National Park (KNP) in South Africa. In KNP, bTB was supposed to spread northwards after introduction of the disease by cattle [50], [51]. Twenty years after the disease was first detected in KNP it is present in most of the park, but prevalences are still much higher in the south [50] and the current distribution of the disease is likely the consequence of that initial infection event, followed by the subsequent northward transmission of the disease from herd to herd. Nevertheless, the spread of disease within buffalo herds in KNP is more recent compared with the situation in DNP, although no precise information is available concerning the evolution in wild ungulates in our study area.

In DNP, cattle are present throughout the wild ungulate range and hence no clear link between cattle presence and wildlife bTB can be drawn. Moreover, the highest prevalences in wildlife are currently reported in a portion of the park that is banned for cattle since 2004. However, it must be taken into account that cattle densities used to be highest in this northern third of DNP prior to cattle exclusion.

Alternative explanations for the observed gradient include sweet water availability, wild ungulate densities (higher in the north than in the south of DNP), habitat characteristics (e.g. [8]), and even genetic factors [10]. Water is more available in summer in the southern portion of the park (MA), as there are numerous waterholes and a long and permanent stretch of spring water along the border of the sand dunes and the marshes. Water availability is minimal in the northern portion of the park, where artificial watering troughs provide drinking water for both domestic and wild animals. Again, more research is needed to clarify whether each of the three zones contains independent sub-populations of ungulates, how they move between zones and how environmental changes could change ungulate dynamics and *M. bovis* transmission patterns.

### Wildlife *M. bovis* reservoirs in Doñana and ecological implications

DNP is a clear case of multi-host system, where all three wild ungulate species, domestic cattle, and to a lesser extent (due to their low densities) carnivores contribute to maintain the circulation of *M. bovis*. Thus, all these species and their environment constitute a huge reservoir in DNP. The importance of the two species of deer in DNP resides in that deer have been suggested to be long-lived reservoirs of infection having the potential to initiate new outbreaks of infection well outside currently infected areas, or to reinitiate infection after TB has been eliminated by controlling reservoirs and other short-lived vectors [33]. However, if one single species is to be highlighted, this is the wild boar due to the prevalence but also to the ecological implications of the species (as scavenger and potential disseminator able to interact with domestic livestock due to its capacity to cross fences). Information gathered in this study contributes to the view that wild boar constitute a true *M. bovis* wildlife reservoir in Mediterranean Spain [7].

Bovine tuberculosis is a multi-host infection with slow progression of disease in the individual. Effects of chronic infectious diseases such as bTB on wildlife population dynamics are often difficult to measure [52]. However, in DNP the lower prevalence observed in adult wild boar as compared to juveniles and yearlings contrasts with figures obtained in hunted populations [5] and suggests that part of the infected juvenile wild boar die due to the disease, as previously suggested in a pathology survey [6]. In deer, the high frequency of generalized bTB found in this survey suggests that bTB may contribute to the periodic die-offs that occur in DNP during severe droughts. Further than prevalence studies, ecological and mortality studies, which probably will require tracking of individuals, are needed to clarify issues such as whether wild boar morbidity has impact on the population's age structure.

Apart from the effects on wild ungulates and the conflict with cattle owners, bTB causes concern regarding the conservation of endangered carnivores such as the Iberian lynx. The high prevalence reported herein in DNP, a core lynx area, makes it highly advisable to consider bTB as a risk factor for this felid, not only in DNP. Broader ecological implications to consider are the probable concomitance and exacerbation of disease due to climatic factors (droughts), and the predation of target animals in poor condition because they are easier to kill by lynxes [53]. The gradient of *M. bovis* infection in ungulates from north to south probably translates into a similar gradient of infection pressure, and this can also determine the risk of spill-over to other species (i. e. Iberian lynx). In this sense, it is known that the highest densities of lynxes in the Park are found in the North area [54].

### Management options for the control of bTB in cattle and wildlife

Wildlife disease control begins with surveillance, knowing if diseases are present, their past and current distribution, and the trends in their prevalence [1]. In DNP, a careful follow up of bTB in both wildlife (mainly in ungulates) and cattle is paramount. *M. bovis* infection was first noticed in the DNP wild ungulate community in the 1980s [17]. In a survey carried out between 1998 and 2003, a 20.5% *M. bovis* infections prevalence was estimated for a non random sample of Doñana wildlife [41]. Current prevalences, as stated above, are among the highest ever reported. In this situation, it cannot be expected that the disease will vanish without strong human intervention. And the compulsory eradication programme for cattle alone has proven to be ineffective.

Attempts to control disease in wildlife populations, or to avoid disease transmission between wildlife and livestock, have been based on a variety of methods. These include setting up barriers, improving hygienic measures, culling, habitat management and feeding bans, treatments and vaccination, among others [1].

Setting up barriers to prevent wildlife contact with domestic livestock is impossible and not necessary in DNP. Perhaps Mostrenca cattle from DNP deserve a special status, considering that bTB eradication is not a realistic goal in the current situation. Movement restrictions for cattle may be a more realistic option.

Hygienic measures, such as correct disposal of carcasses and carcass remains as it is done in DNP should become compulsory in other areas, particularly in Iberian lynx habitats. However, research on such management is needed in favour of the conservation of carrion consuming endangered bird species.

The identification and correction of overabundance situations are key actions in the control of many infectious diseases [55]. While wildlife culling is almost never an effective means to eradicate a wildlife-related disease, and it is a subject of intense scientific and social debate (e.g. badger culling for bTB control [56]), population reduction is a goal in many disease control efforts. This is a temporary measure, except if habitat modification is used to reduce host density more permanently or to alter host distribution or exposure to disease agents [9], [55], [57]. In DNP, a realistic though arbitrary goal would be to achieve a 50% reduction of the current wild ungulate density, and efforts should be biased towards wild boar and red deer, since these species show higher prevalence than fallow deer. Observational data from central Spain have shown a relationship among wild boar abundance, wild boar aggregation, and bTB prevalence [9]. A correlation between wild boar and red deer bTB prevalence was also shown in the same region [5].

The success of any such management action must be assessed critically, including an analysis of the costs, of the ecological consequences and of the animal and human health and welfare benefits. At the very least, wild boar and the two deer species in Doñana are not endangered species. They can be hunted within the Park under special permits, and their populations may even be reinforced or substituted, if that was deemed necessary, with quarantined individuals from other areas.

We recommend continuing park-wide monitoring activities including different areas, with special emphasis on the cattle free northern third of DNP to understand disease dynamics and risks, ad hoc surveillance for *M. bovis* infection in all possible species which could play a role in TB epidemiology, identifying and culling of animals with advanced bTB to reduce the number of super-shedders and the availability of contagious carrion. Also, future evaluation of new vaccine candidates and investigating genetic markers for resistance to intracellular bacteria may contribute to control the infection. All these actions must be faced in parallel with studies elucidating the ecological impact of bTB in DNP wildlife.
